# T109. CLUSTERING OF SCHIZOPHRENIA PATIENT SUBTYPES BY SPECIFIC SYMPTOM DIMENSIONS USING AN UNCORRELATED PANSS SCORE MATRIX (UPSM)

**DOI:** 10.1093/schbul/sby016.385

**Published:** 2018-04-01

**Authors:** Seth Hopkins, Ajay Origala, Antony Loebel, Kenneth S Koblan

**Affiliations:** Sunovion Pharmaceuticals

## Abstract

**Background:**

Interpretation of the efficacy of antipsychotic agents in treating schizophrenia using standard (Marder) Positive and Negative Syndrome Scale (PANSS) factors is confounded by moderate-to-high between-factor correlations. In previous pooled analyses of short-term, placebo-controlled lurasidone clinical trials, clustering and factor analysis identified an uncorrelated PANSS score matrix (UPSM) that generated transformed PANSS factor scores with high face validity (good correlation with standard [Marder] PANSS factors), and high specificity/orthogonality (low levels of between-factor correlation) at both baseline, and when measuring change during short-term treatment. In a validation analysis using 12 separate clinical trials, we previously confirmed that the weighted UPSM coefficients had generalizable utility, yielding transformed PANSS factors with high specificity while retaining good levels of correlation with standard PANSS factors. The aim of the current analysis was to determine whether distinct clinical subtypes of schizophrenia could be empirically derived from the transformed PANSS factor scores at baseline.

**Methods:**

In a new analysis of a pooled sample of 5 placebo-controlled trials (N=1,710 patients), K-means clustering of baseline UPSM factor scores in MATLAB was used to identify whether clinical sub-groups could be empirically derived that were characterized by predominant symptom severity in one or more of the transformed PANSS factor domains. For each empirically derived domain thus identified, key demographic and clinical variables were examined, including baseline transformed PANSS factor severity scores [note: the weighted UPSM coefficient yields factor scores with numerical values that are much smaller than are observed with standard Marder factor scores]; and Montgomery-Åsberg Depression Rating Scale (MADRS) and Negative Symptom Assessment Scale (NSA) scores.

**Results:**

Cluster analysis using the UPSM transformed PANSS Factor scores identified 5 distinct clinical subtypes defined by the severity of the UPSM Factor score relative to the mean score for all patients on the respective transformed PANSS factors. For the predominant positive cluster, the mean transformed PANSS positive factor score was 3.9 (vs. a mean score of 2.9 ± 0.9 SD for all patients); for the predominant hostile cluster, the hostility factor score was 2.6 (vs. a mean score of 1.4 ± 1.1); for the predominant disorganized cluster, the disorganized factor score was 3.0 (vs. 2.5 ± 1.0); for the affective cluster, the anxiety and depression factors, respectively, were 2.3 (vs. 1.8 ± 0.9) and 2.7 (vs. 1.7 ± 1.0); and for the predominant negative cluster, the apathy/avolition and deficit of expression factors, respectively, were 3.1 (vs. 2.5 ± 0.9) and 2.5 (vs. 1.8 ± 0.9). Patients in the predominant negative cluster had the highest NSA score (61 vs. a mean score overall of 53); and patients in the predominant affective cluster had the highest MADRS score (16 vs. a mean score overall of 11).

**Discussion:**

These results provide evidence for a consistent underlying schizophrenia symptom structure and suggest the utility of UPSM transformed PANSS factors for characterizing clinical differences among clearly delineated clinical subpopulations, even within a clinical trial population of acute schizophrenia.

